# Cytotoxic and Antioxidant Activity of *Hypericum perforatum* L. Extracts against Human Melanoma Cells from Different Stages of Cancer Progression, Cultured under Normoxia and Hypoxia

**DOI:** 10.3390/molecules28031509

**Published:** 2023-02-03

**Authors:** Aleksandra Brankiewicz, Sara Trzos, Magdalena Mrożek, Małgorzata Opydo, Elżbieta Szostak, Michał Dziurka, Monika Tuleja, Agnieszka Łoboda, Ewa Pocheć

**Affiliations:** 1 Department of Plant Cytology and Embryology, Institute of Botany, Faculty of Biology, Jagiellonian University, 30-387 Kraków, Poland; 2 Department of Glycoconjugate Biochemistry, Institute of Zoology and Biomedical Research, Faculty of Biology, Jagiellonian University, 30-387 Kraków, Poland; 3 Laboratory of Experimental Hematology, Institute of Zoology and Biomedical Research, Faculty of Biology, Jagiellonian University, 30-387 Krakow, Poland; 4 Faculty of Chemistry, Jagiellonian University, 30-387 Kraków, Poland; 5 The Franciszek Górski Institute of Plant Physiology, Polish Academy of Sciences, 30-239 Kraków, Poland; 6 Department of Medical Biotechnology, Faculty of Biochemistry, Biophysics and Biotechnology, Jagiellonian University, 30-387 Kraków, Poland

**Keywords:** *Hypericum perforatum* L., primary and metastatic melanoma, cytotoxicity, antioxidant activity, apoptosis, normoxia, hypoxia, hyperforin

## Abstract

Oxidative stress and the hypoxic microenvironment play a key role in the progression of human melanoma, one of the most aggressive skin cancers. The aim of our study was to evaluate the effect of *Hypericum perforatum* extracts of different origins (both commercially available (HpEx2) and laboratory-prepared from wild grown (HpEx12) and in vitro cultured (HpEx13) plants) and hyperforin salt on WM115 primary and WM266-4 lymph node metastatic human melanoma cells cultured under normoxic and hypoxic conditions. The polyphenol content, radical scavenging activity, and hyperforin concentration were determined in the extracts, while cell viability, apoptosis, ROS production, and expression of NRF2 and HO-1, important oxidative stress-related factors, were analyzed after 24 h of cell stimulation with HpExs and hyperforin salt. We found that cytotoxic, pro-apoptotic and antioxidant effects depend on the extract composition, the stage of melanoma progression, and the oxygen level. Hyperforin salt showed lower activity than *H. perforatum* extracts. Our study for the first time showed that the anticancer activity of *H. perforatum* extracts differs in normoxia and hypoxia. Importantly, the composition of extracts of various origins, including in vitro cultured, resulting in their unique properties, may be important in the selection of plants for therapeutic application.

## 1. Introduction

Cutaneous melanoma (CM) is skin cancer originating from melanocytes, neural crest-derived cells responsible for protecting the skin against UV radiation, that can simultaneously be precursors of these malignant cancer cells [1,2]. The incidence rate of CM depends on genetic factors, but also geographical location and risk factors, such as UVA and UVB exposure without skin protection [2]. Despite the low incidence (1–3%) among skin cancers, this form is the deadliest [3,4,5]. Although the primary tumor can be treated successfully with surgery, metastatic melanoma poses a serious challenge for therapy and is highly lethal [6]. Due to its metastatic capacity, CM is one of the main causes of cancer mortality. In recent decades, there have been some advances in the treatment of malignant CM, involving primarily RAF and MEK kinase inhibitors, as well as immune antibody-mediated checkpoint inhibitors, the approaches of targeted therapy [2,7]. However, despite significant progress, new therapeutic strategies are required due to difficulties in matching therapy with the patient and tumor resistance to treatment [8].

Oxidative stress is an important factor involved in the regulation of cancer metabolism and can lead to cell progression or cell death. A high level of reactive oxygen species (ROS) activates the antioxidant transcription factors responsible for, e.g., cell protection [9]. One of these transcription factors is the nuclear factor erythroid-like 2 (NRF2, encoded by *NFE2L2*), inactivated by the Kelch-like protein ECH-associated protein 1 (KEAP1) repressor, and directed to proteasomal degradation under normal conditions (no oxidative stress factors). In contrast, during oxidative stress, the repressor is modified by electrophiles at cysteine positions, and NRF2 is phosphorylated, resulting in a lack of transcription factor degradation and raising its level in the nucleus. Under conditions of intensified oxidative stress, NRF2, through binding to a region called the antioxidant response element (ARE), regulates the expression of approximately 250 genes encoding anti-inflammatory, antioxidant, and detoxifying proteins [10]. Among these genes, up-regulated by NRF2, is heme oxygenase 1 (HO-1), the inducible isoform of this protein, which controls cell proliferation, angiogenesis, inflammation, apoptosis, and the rate of heme degradation, which can reduce the risk of intracellular ROS generation [11]. In cancers, NRF2 and HO-1 are of great importance. Despite the initial recognition of NRF2 as a target for chemopreventive compounds, this transcription factor can provide enhancement and inhibition of carcinogenesis [10,12]. Interestingly, HO-1 can promote tumor metastasis and be overexpressed during treatment [13]. However, the role of HO-1 in carcinogenesis depends on the tumor type, and proper induction of its level may be a therapeutic strategy. Some reports indicate the positive role of plant extracts in cancer therapy by inducing the NRF2/HO-1 pathway activation, suggesting its role in other than apoptosis cell death types, e.g., ferroptosis [14]. Alongside oxidative stress, hypoxia is also a factor that affects cancer cell functioning. Violent proliferation can cause tumor overgrowth and, consequently, a drastic drop in oxygen concentration even below 2%, caused by vascular insufficiency. Hypoxic regions of tumor tissue can be more drug resistant than cells in a higher oxygen concentration, and, in addition, these regions may be the mainstay of cancer stem cells that promote epithelial–mesenchymal transition (EMT) and metastasis [15]. Therefore, hypoxic conditions are extremely important in analyzing the cytotoxicity of drugs selected for cancer treatment.

Damaged normal cells with mutations are directed to apoptosis, while cancer cells are apoptosis resistant. Therefore, the induction of apoptosis is one of the most important targets for cancer treatment. During apoptosis, chromatin condensation, DNA fragmentation, and consequently, apoptotic body formation, are observed. A key role in this process is played by caspases, a cysteine protease activated in an extrinsic or intrinsic apoptotic signaling pathway [16]. Certain plant extracts have been shown to contain compounds, especially polyphenols, that promote apoptosis [17,18]. Importantly, plant-derived substances cause less side effects than conventional chemotherapeutics; moreover, many of them have chemopreventive properties, so they are worth considering as anti-cancer drugs [19].

*Hypericum perforatum* L. (St. John’s wort) is a perennial herb widespread in Europe, and one of the most valued traditional and pharmacological agents grown on plots and harvested from natural sites [20]. Its anti-microbial, anti-depressant, antioxidative, anti-inflammatory and anti-cancer properties have been proven due to the content of numerous secondary metabolites (SM), and among them are polyphenols [21,22], with flavonoids and biflavonoids, xanthones, phenylpropanes, and except for phenolics, two main *Hypericum* compound classes: hypericins and hyperforins [23]. Most reports indicate that alcoholic extracts of *H. perforatum* exert antioxidant and anti-proliferative properties [24,25]. Hyperforin, a polyprenylated acylphloroglucinol derivative extracted from *H. perforatum*, exhibits numerous activities, such as antidepressant, antibiotic against Gram-positive bacteria, and anti-cancer properties. It can lead to the induction of intrinsic and extrinsic apoptotic pathways, and the inhibition of human malignant tumor cell growth [26,27,28,29].

Although the anti-cancer effects of *H. perforatum* extracts have been demonstrated before, this issue is still not fully addressed. It is especially unclear whether the response of cancer cells at different stages of progression is comparable to the active components of extracts, and how the oxygen level, decreased inside tumors, affects the anti-cancer activity of extracts. Therefore, the aim of this study was to evaluate the effects of ethanolic extracts of different origins, commercially available, and laboratory-prepared, in vitro and wild grown *H. perforatum*, on primary (WM115) and metastatic (WM266-4) CM cell lines under normoxic and hypoxic conditions. We have focused on the comparison of extracts and the effect of hyperforin dicyclohexylammonium salt on melanoma cell viability, apoptosis, and oxidative stress.

## 2. Results

### 2.1. Characteristics of Hypericum perforatum Ethanolic Extracts

Among a set of *H. perforatum* ethanolic extracts (HpExs; 11 laboratory-prepared and two commercially available, tested in the preliminary stage of the study, data not shown), three HpExs were finally chosen to analyze their cytotoxic, proapoptotic, and antioxidant activities. A selected commercial extract (HpEx2) was obtained from Herbapol (Poland), and two extracts were self-made, HpEx12 was prepared from two wild populations of *H. perforatum* located in Krakow (Poland) and HpEx13 from in vitro regenerants obtained by indirect organogenesis in explants derived from the same populations. Furthermore, the effect of hyperforin salt was also evaluated, as its anti-cancer activity has previously been well documented [24,25,26,28,29].

The content of the most important SM for this study, selected based on previous research [21,22,23], was determined in the extracts. The amount of hyperforin in HpExs was analyzed by HPLC-MS/MS, and the content of polyphenols was measured by a colorimetric TPC test, using gallic acid as a standard. The self-prepared extracts contained approximately twice less ethanolic-dissolved dry substances than the Herbapol product, but both, commercially available HpEx2, and laboratory-prepared HpEx12 and HpEx13, showed approximately 8–10% of phenolic content in the dry extract (Table 1). Importantly, commercial and self-made extracts differed strongly in hyperforin content, in favor of the extract obtained from wild-type plants, especially in vitro-derived regenerants (HpEx13), having over 130x more this chemical than Herbapol’s product (HpEx2), and almost 1.5x more than the extract from the same two *H. perforatum* wild populations (HpEx12).

After bringing the extracts to a concentration of 3 mg/mL, the strongest radical scavenging capacity measured in the DPPH assay (the lowest half-maximal inhibitory concentration, IC50) was obtained for the commercial extract, and slightly lower capability (higher IC50) was detected for extracts of wild-harvested and in vitro cultured *H. perforatum* (Table 1). Hyperforin salt did not demonstrate free radical scavenging properties (data not shown).

### 2.2. Hypericum perforatum Extracts and Hyperforin Salt Affect Melanoma Cell Viability in an Oxygen-Dependent Manner

**Figure 1:**
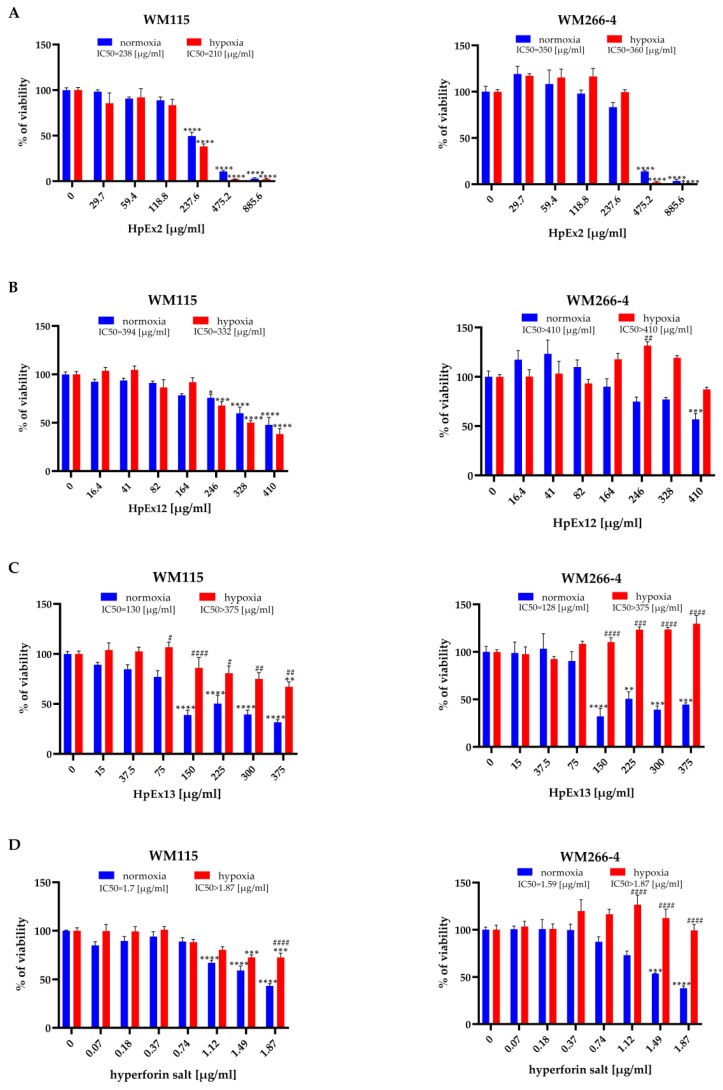
Effects of *Hypericum perforatum* extracts (**A**–**C**) and hyperforin salt (**D**) on the viability of the WM115 and WM266-4 human melanoma cell lines cultured under normoxia (blue bars) and hypoxia (red bars) conditions, determined by the MTT assay. Results are expressed as mean values ± SD. IC50 values are assigned above the chart legends. Statistical significance of the data was assessed using a two-way ANOVA, followed by a Tukey’s test for an honest significant difference over multiple ranges. Significance levels between treated cells relative to untreated cells are marked with asterisks as follows: * *p* ≤ 0.05; ** *p* ≤ 0.01; *** *p* ≤ 0.001; **** *p* ≤ 0.0001. Comparisons for treated cells within a given dose between normoxia and hypoxia are marked with hash marks as follows: # *p* ≤ 0.05; ## *p* ≤ 0.01; ### *p* ≤ 0.001; #### *p* ≤ 0.0001.

To test how the active substances present in *H. perforatum* extracts and hyperforin salt affect the viability of WM115 primary and WM266-4 metastatic melanoma cells in normoxia and hypoxia, the MTT assay was performed, and the IC50 values of each HpEx and hyperforin salt were determined (Figure 1). The Hs27 human skin fibroblast cell line was also used to assess the cytotoxicity of HpExs 2, 12, and 13 on normal cells cultured under normoxia (Supplementary Materials Figure S1).

The results obtained showed significant differences in the cytotoxic effect of HpExs between primary and metastatic melanoma cells, and also between normoxia and hypoxia within analyzed cell lines. The viability of primary WM115 cells treated with HpExs and hyperforin salt showed a dose-dependent decrease under both oxygen conditions (except HpEx13 in hypoxia, as only the highest concentration reduced cell viability). In contrast, the reduction of the WM266-4 metastatic cell viability was observed only in normoxia, except for HpEx2 in the highest concentrations (475.2 μg/mL and 885.6 μg/mL), with a strong cytotoxic effect also in hypoxia. What is especially interesting is that the highest doses of HpEx12, hyperforin salt, and, in particular, HpEx13, activated the viability and/or metabolic activity of WM266-4 metastatic cells in hypoxia (Figure 1).

The various effects of commercial HpEx2 in comparison to laboratory-prepared extracts 12 and 13 on the viability of melanoma cells may result from their different compositions (Table 1, Supplementary Materials Table S1). The content of polyphenols in the HpEx2 volume added to the cells was approximately twice that of the two other extracts, while the amount of hyperforin, the highest in HpEx13, was almost 50 times lower in HpEx2.

It is should be noted that a weak response from Hs27 fibroblasts to active ingredients and SM was observed in HpEx13, and no effect was noticed in the case of HpEx12. HpEx2 with the highest content of polyphenols impaired the viability of Hs27 cells more significantly than the two other extracts, but its IC50 value for Hs27 cells (680 μg/mL) was significantly higher (Supplementary Materials Figure S1) than for primary (238 μg/mL) and metastatic (350 μg/mL) melanoma cells in normoxia (Figure 1A).

### 2.3. Hypericum perforatum Extracts and Hyperforin Salt Have a Pro-Apoptotic Effect on Melanoma Cells

**Figure 2:**
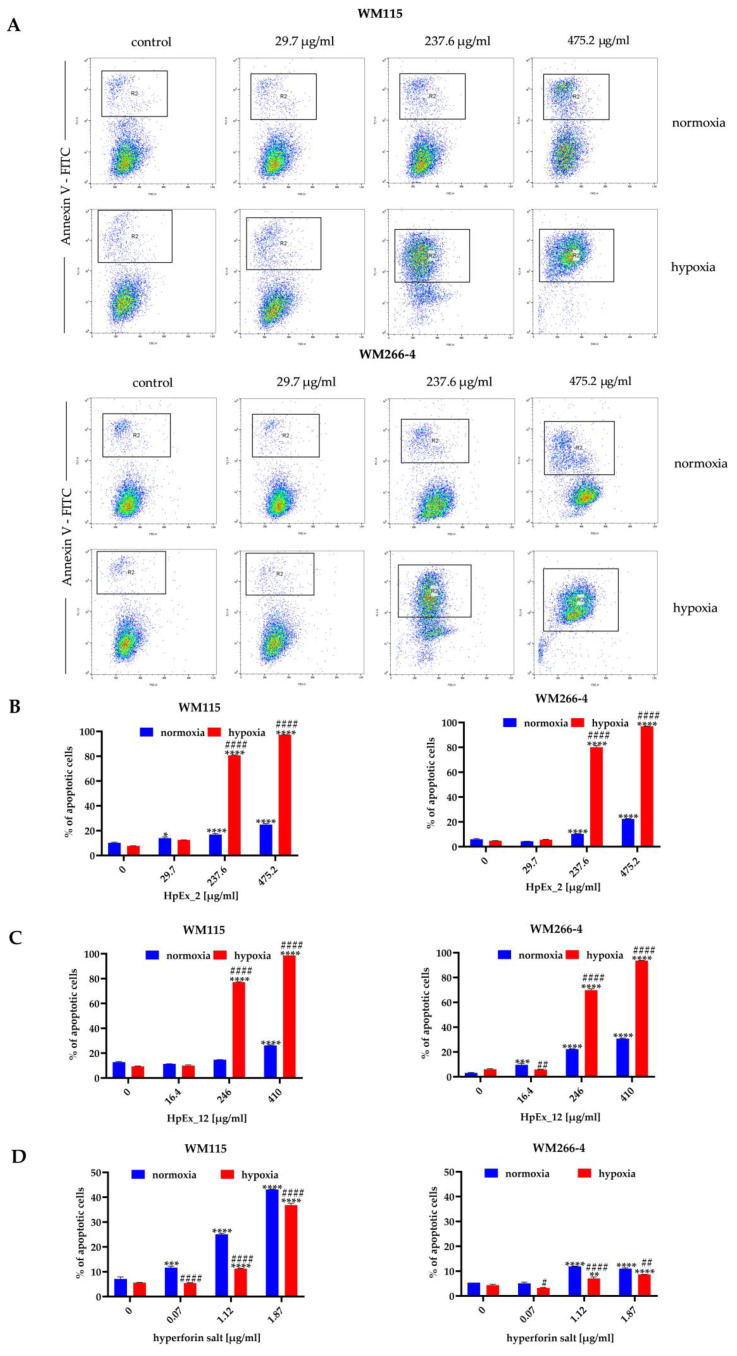
Detection of apoptosis in the WM115 and WM266-4 melanoma cells treated with ethanolic extracts of *Hypericum perforatum* and hyperforin salt, cultured under normoxia (blue bars) and hypoxia (red bars), determined by FITC-conjugated Annexin V staining in flow cytometry. Representative dot plots for WM115 and WM266-4 cells treated with commercially available extract 2 (HpEx2) (**A**), and bar charts for melanoma cells treated with HpEx2 (**B**), HpEx12 (**C**), and hyperforin salt (**D**). The results are expressed as mean values ± SD. Statistical significance for the data was evaluated by a two-way ANOVA, followed by a Tukey’s honestly significant difference, multiple range test. Significance levels between treated cells relative to untreated cells are indicated with asterisks as follows: * *p* ≤ 0.05; ** *p* ≤ 0.01; *** *p* ≤ 0.001; **** *p* ≤ 0.0001. The comparison for treated cells within a given dose between normoxia and hypoxia is indicated with hash marks as follows: # *p* ≤ 0.05; ## *p* ≤ 0.01; #### *p* ≤ 0.0001.

**Figure 3:**
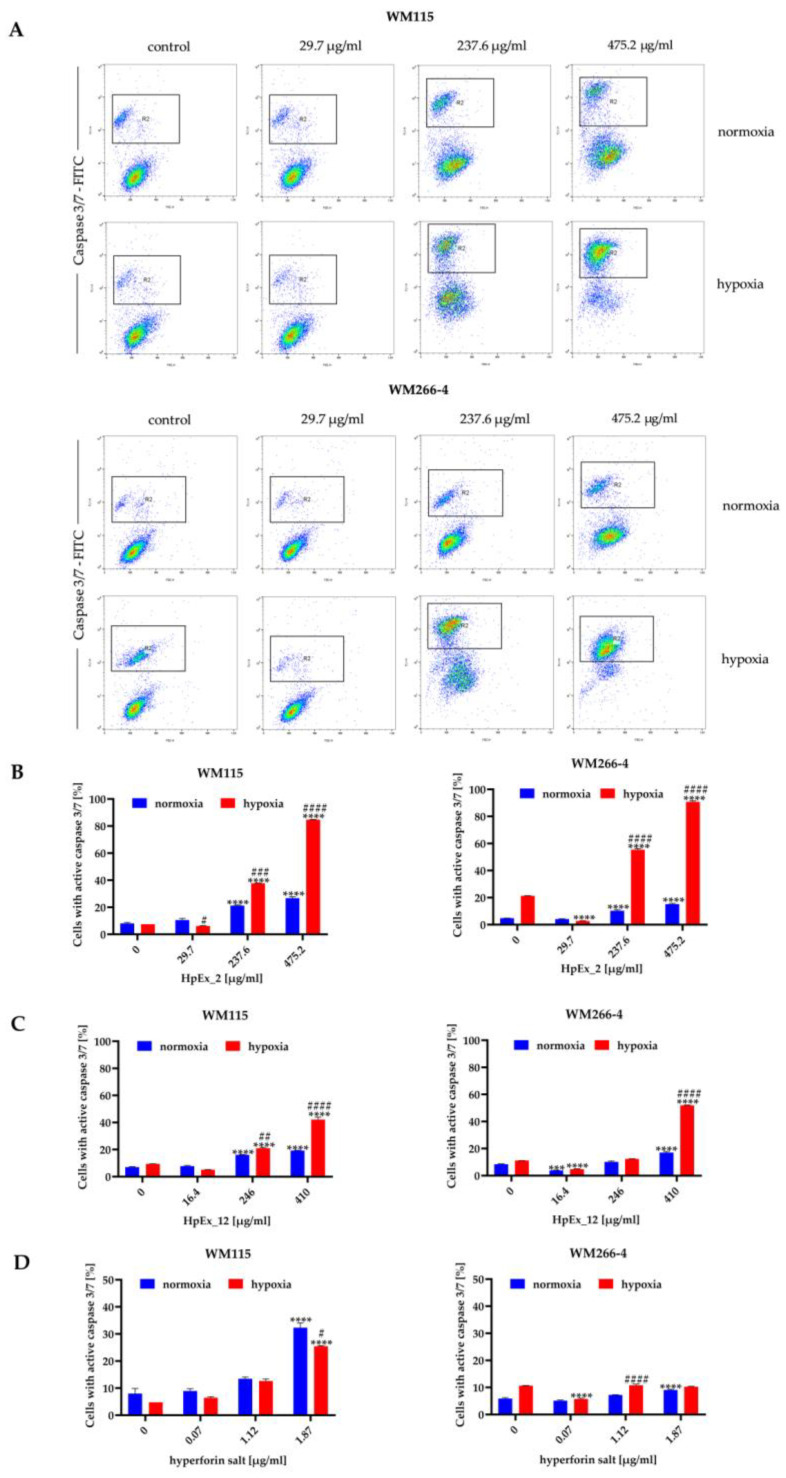
Detection of activated caspases 3 and 7 in apoptotic cells of the WM115 and WM266-4 melanoma lines treated with ethanolic extracts of *Hypericum perforatum* and hyperforin salt, cultured under normoxia and hypoxia, determined by flow cytometry. Representative dot plots for WM115 and WM266-4 cells treated with commercially available extract (HpEx2) (**A**), and bar charts for melanoma cells treated with HpEx2 (**B**), HpEx12 (**C**), and hyperforin salt (**D**). The results are expressed as mean values ± SD. Statistical significance for the data was evaluated by a two-way ANOVA, followed by a Tukey’s honestly significant difference, multiple range test. Significance levels between treated cells relative to untreated cells are indicated with asterisks as follows: ** *p* ≤ 0.01; *** *p* ≤ 0.001; **** *p* ≤ 0.0001. The comparison for treated cells within a given dose between normoxia and hypoxia is indicated with hash marks as follows: # *p* ≤ 0.05; ## *p* ≤ 0.01; ### *p* ≤ 0.001; #### *p* ≤ 0.0001.

The decrease in cell viability in the presence of HpExs and hyperforin salt, measured in the MTT assay (Figure 1), may be the result of reduced cellular metabolic activity and proliferation, as well as increased apoptosis. The proapoptotic activity of plant-derived substances is especially desirable for cancer treatment; therefore, in the next stage of our study, the possible induction of apoptosis by HpExs and hyperforin salt was determined using the annexin V (Figure 2) and activated caspase 3 and 7 (Figure 3) tests.

The results obtained in both apoptosis assays demonstrated a dose- and oxygen-dependent response from melanoma cells to extracts 2 and 12, and hyperforin salt. The ability to induce apoptosis was comparable between both extracts, and a significantly stronger effect was observed for both HpExs extracts in hypoxia than normoxia (Figure 2B,C and Figure 3B,C). Hyperforin salt also exerted an apoptotic effect on melanoma cells, but WM266-4 metastatic cells showed significantly greater resistance to this compound than WM115 primary cells (Figure 2D and Figure 3D). The most pronounced effect was observed for WM115 cells treated with hyperforin salt at the concentrations of 1.12 μg/mL and 1.87 μg/mL, for which 14% and 33% (normoxia), and 12% and 26% (hypoxia) of the primary melanoma cells showed externalization of phosphatidylserine, respectively, while no more than 10% of WM266-4 metastatic cells showed the features of apoptosis under the same conditions (Figure 2D).

Hyperforin salt doses were selected based on the hyperforin content in HpEx12 volumes used in cell tests (Supplementary Materials Table S1). Despite the standardized content of hyperforin, the percentage of apoptosis within hypoxia-cultured melanoma cells treated with hyperforin salt was significantly lower than in the case of HpEx12, as shown in the annexin V and caspase3/7 assays. In turn, WM115 primary melanoma cells cultured in normoxia were more susceptible to apoptosis in the presence of hyperforin salt than HpEx12. The diverse response of melanoma cells to hyperforin salt and HpEx12, with the same content of hyperforin, may result from the synergistic effect of SM in the extract. The oxygen level is also crucial for the final apoptotic effect of the examined compounds.

### 2.4. Hypericum perforatum Extracts Reduce the Level of Reactive Oxygen Species in Melanoma Cells

**Figure 4:**
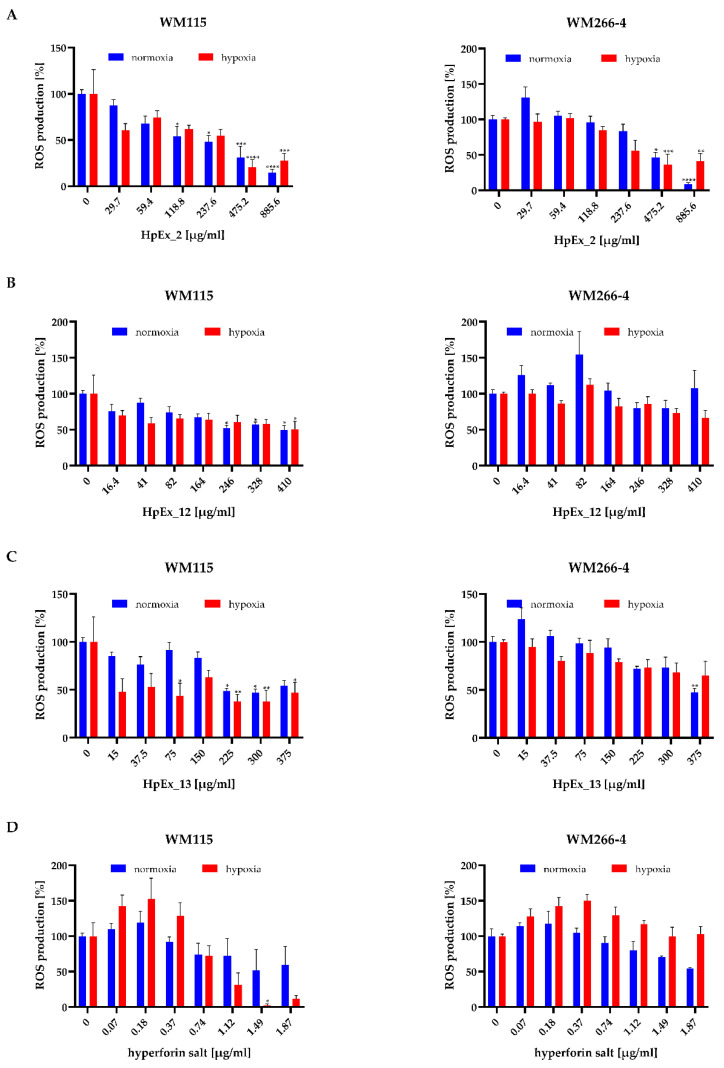
Effect of *Hypericum perforatum* extracts HpEx2 (**A**), HpEx12 (**B**), Hpx13 (**C**) and hyperforin salt (**D**) on ROS production by WM115 and WM266-4 melanoma cells cultured under normoxia (blue bars) and hypoxia (red bars), determined by the NBT assay. Results are expressed as mean values ± SD. The statistical significance of the data was assessed using a two-way ANOVA, followed by a Tukey’s test for a fair significant difference over multiple ranges. Significance levels between treated cells relative to untreated cells are indicated with asterisks as follows: * *p* ≤ 0.05; ** *p* ≤ 0.01; *** *p* ≤ 0.001; **** *p* ≤ 0.0001.

Due to the importance of oxidative stress in melanoma progression, in the next stage of our research, the impact of HpExs and hyperforin salt on ROS generation was assessed. (Figure 4).

The results obtained for HpExs, shown in Figure 4A–C, indicate a statistically important decrease in ROS generation in melanoma cells cultured under normoxia and hypoxia by extracts administered at the highest concentrations, with the strongest effect found for HpEx2. After exposure of melanoma cells to HpEx2, a significant reduction in oxidative stress was observed for the two highest doses, 475.2 μg/mL and 885.6 μg/mL, where the ROS level decreased between 50% and 15% under both oxygen conditions (Figure 4A). The activity of HpEx12 attenuated ROS generation in a statistically significant way only in WM115 primary cells at the concentrations of 246 μg/mL, 328 μg/mL, and 410 μg/mL in normoxia, and 410 μg/mL in hypoxia, while WM266-4 metastatic cells were not affected by this extract (Figure 4B). HpEx13 was also more effective for the primary than metastatic melanoma cells, and caused a statistically significant decrease in ROS generation in the WM115 cells under both oxygen conditions for dose ranges between 225–375 μg/mL in normoxia and 75–375 μg/mL in hypoxia, while in WM266-4 cells, only the highest concentration of 375 μg/mL under normoxia had antioxidant activity (Figure 4C). It is worth noting that the differences in ROS generation after stimulation of melanoma cells with commercial (HpEx2) and laboratory-made (HpEx12 and 13) extracts may be due to their different compositions, where HpEx2 contains the highest amount of polyphenols, while the extract from in vitro cultured plants HpEx13 shows a high content of hyperforin (Table 1, Supplementary Materials Table S1).

ROS levels in both melanoma cell lines were lower with increased doses of hyperforin salt, but the statistically important difference was found for only one of the highest concentrations for WM115 primary cells in hypoxia. ROS production in the presence of hyperforin salt was higher in the metastatic cell line than in primary melanoma cells, and was more intense in WM266-4 cells cultured at 0.5% O_2_ than at 21% O_2_ (Figure 4D).

### 2.5. Hypericum perforatum Extracts and Hyperforin Salt Alter the Expression of NRF2 and HO-1 on Gene and Protein Levels

**Figure 5:**
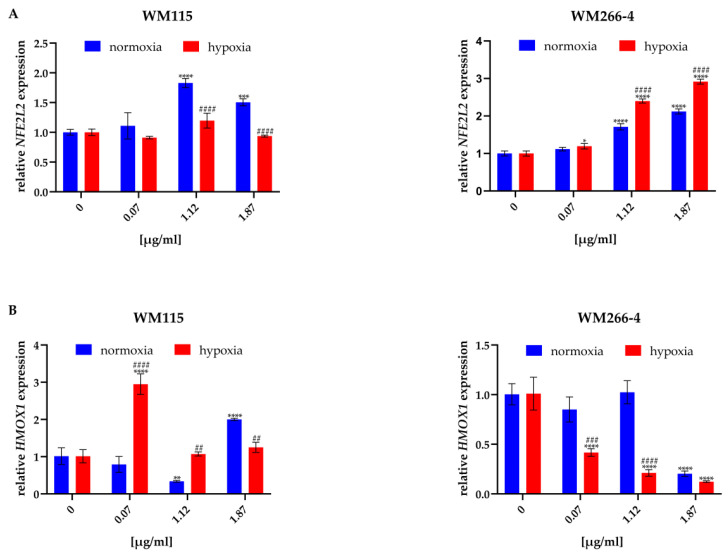
Effect of hyperforin salt on *NFE2L2* (**A**) and *HMOX1* (**B**) gene expression in WM115 and WM266-4 melanoma cells cultured under normoxia (blue bars) and hypoxia (red bars), determined by RT-qPCR. Results are expressed as mean values ± SD. The statistical significance of the data was assessed using a two-way ANOVA, followed by a Tukey’s test for a fair significant difference over multiple ranges. Significance levels between treated cells versus untreated cells are indicated by asterisks as follows: * *p* ≤ 0.05; ** *p* ≤ 0.01; *** *p* ≤ 0.001; **** *p* ≤ 0.0001. Comparisons for treated cells within a given dose between normoxia and hypoxia are indicated by hashes as follows: ## *p* ≤ 0.01; ### *p* ≤ 0.001; #### *p* ≤ 0.0001.

**Figure 6:**
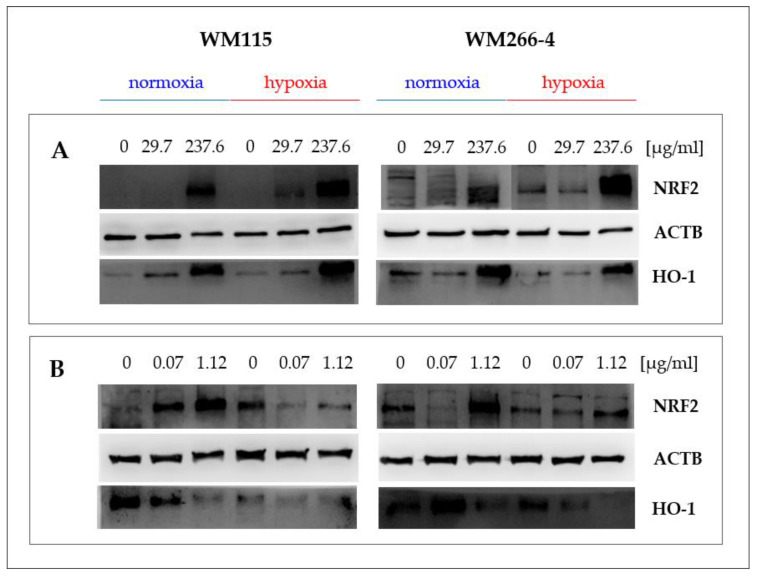
Effect of *Hypericum perforatum* extract HpEx2 (**A**) and hyperforin salt (**B**) on NRF2 and HO-1 protein expression in WM115 and WM266-4 melanoma cells cultured under normoxia and hypoxia, analyzed by the specific anti-NRF2 (16396-1-AP, Proteintech) and anti-HO-1 (ADI-SPA 894, Enzo) antibodies in Western blotting.

NRF2/HO-1 signaling pathway is crucial in the regulation of oxidative stress [11], therefore, in the last part of the study we analyzed the impact of *H. perforatum* extracts and hyperforin salt on NRF2 and HO-1 expression at gene (Figure 5) and protein (Figure 6) levels. The concentrations of HpExs and hyperforin salt for these experiments were chosen based on the results obtained in the MTT cell viability assay (Section 2.2).

The expression of the *NFE2L2* and *HMOX1* genes, encoding the NRF2 and HO-1 proteins, respectively, was analyzed only for the melanoma cells treated with hyperforin salt, due to the problem with the purity of RNA isolated from cells treated with HpExs, rich in phenolic compounds. To quantify how 24 h exposure of melanoma cells to hyperforin salt in normoxia and hypoxia affects the expression of the genes mentioned above, RT-qPCR analysis was performed in relation to the expression of *HPRT*, a gene encoding hypoxanthine phosphoribosyltransferase 1, stably expressed under the tested conditions.

Treatment of melanoma cells cultured under normoxia and hypoxia with hyperforin salt significantly altered the expression of both oxidative stress-related genes (Figure 5). Hyperforin salt increased the expression of *NFE2L2* in a dose-dependent manner and changed the expression of *HMOX1* in the primary cells of WM115. In WM266-4 metastatic cells, an enhancement of *NFE2L2* expression, accompanied by an attenuation of *HMOX1* level in normoxia and hypoxia, was found.

Differences between normoxia and hypoxia were also observed within a given dose of hyperforin salt (1.12 μg/mL and 1.87 μg/mL). In WM115 cells, the increase in *NFE2L2* expression was lower under hypoxic than normoxic conditions, in contrast to WM266-4 cells, in which hypoxia promoted its expression (Figure 5A). The expression of *HMOX1* was lower in hypoxia than in normoxia in WM266-4 cells treated with hyperforin salt at all concentrations and in WM115 cells at the highest concentration of this SM. In the presence of 0.07 μg/mL and 1.12 μg/mL of hyperforin salt, the *HMOX1* level was significantly up-regulated in WM115 cells cultured under hypoxia (Figure 5B).

The content of NRF2 and HO-1 proteins in melanoma cells cultured in normoxia and hypoxia in the presence of extracts 2 and 12, and hyperforin salt, was analyzed by Western blotting, using specific anti-NRF2 and anti-HO-1 antibodies (Figure 6).

We observed a significant increase in NRF2 and HO-1 levels in the primary and metastatic cells cultured in the presence of the highest concentration of extracts HpEx2 (237.6 μg/mL, Figure 6A) and HpEx12 (246 μg/mL, data not shown) under both oxygen conditions. The most varied response in the expression of NRF2 and HO-1 proteins was found for hyperforin salt (Figure 6B). In both melanoma cell lines cultured under normoxia, an increase of NRF2 level was accompanied by a decrease of HO-1 content in the presence of hyperforin salt applied in the concentrations of 0.07 μg/mL and 1.12 μg/mL. In hypoxia, changes in the amount of the analyzed proteins triggered by hyperforin salt were different for primary and metastatic cells. Both NRF2 and HO-1 levels were down-regulated in WM115 cells treated with hyperforin salt, while in WM266-4 cells, NRF2 content was increased and HO-1 reduced in the presence of this SM. The expression of the *NFE2L2* and *HMOX1* genes (Figure 5) correlates with the content of the NRF2 and HO-1 proteins (Figure 6B) in melanoma cells treated with hyperforin salt, except for WM115 cells cultured in hypoxia. It may be due to a time change in gene and protein expression that is likely different for primary and metastatic cells with various proliferation rates, especially under oxygen-limited conditions.

## 3. Discussion

Nowadays, the public interest in the use of extracts obtained from raw plant materials as anticancer agents, or isolated plant extract ingredients combined with drugs, is constantly growing, particularly due to the increase of cancer incidence cases and tumor drug resistance [19,30,31]. The raw material of *Hypericum perforatum* (*Hyperici herba*) is rich in variable chemicals, including, among others, phenolics and flavonoids, which are typical of compounds of the genus *Hypericum*, with hyperforin and its derivatives. Hyperforin, widely known as an antidepressant agent [32,33], has also confirmed an anticancer effect [26,34]. Considering the wide range of its occurrence in central Europe and Asia, and its commonness, *H. perforatum* is potentially cheap to obtain and easily available for research in phytochemistry studies, despite its chemical content being dependent on environmental conditions [20,21,35]. The reported antiproliferative, cytotoxic, proapoptotic and antioxidant properties of hyperforin [36] led researchers to improve the methods of its extraction of its raw material from plants to increase the sourcing efficiency [37].

Our study was designed to determine the activity of *H. perforatum* extracts of various origins with different compositions of polyphenols and hyperforin against primary and metastatic melanoma cells under normoxic and hypoxic conditions. The salient finding of our study is that the anticancer action of the *H. perforatum* extracts differs in normoxia and hypoxia, which is especially important due to the changes in oxygen levels in tumor growth and progression.

### 3.1. Wild-Grown and In Vitro Cultured Hypericum perforatum Extracts Differ in Composition from Commercial Ethanolic Extract

The experimental studies of our work focused on verifying the effect of commercially available hyperforin salt, and comparing them with the effect of fully-composed *H. perforatum* ethanolic extracts on melanoma cell lines from various stages of progression (primary WM115 and metastatic WM266-4). To verify the effectiveness of extracts, we have performed screening research to compare the cytotoxicity and antioxidant activity with the chemical composition of extracts, taking into account the hyperforin and total phenolic content in the dry mass. Obtained results reveal differences in hyperforin content between the three extracts used. HpEx2 contains trace amounts of hyperforin, compared to HpEx12 and HpEx13, with larger amount of this chemical. Conversely, the highest content of phenolic compounds was noted in HpEx2, which proves the influence of *H. perforatum* origin and cultivation way on the chemical composition, which was also confirmed in other experimental works [38,39]. The quality of *Hypericum* raw material depends, for example, on altitude [39], genotype [40], species fertilization scheme, cultivation [20], microbiome and chemical factors [23,41], extraction procedure, solvent, collection date, and storage of raw material [42]. The significance of environmental and genotypical factors affecting hyperforin content prompted us to regenerate harvested *H. perforatum* in in vitro cultures to verify hyperforin production by St John’s wort under natural and laboratory conditions. Revealed differences in hyperforin content between the commercially available extract and wild-origin harvested plants shed new light on the extraction of hyperforin from St. John’s wort growing in natural populations. However, to protect the genetic variability of this species from natural sources, the better solution is to use natural plant cell totipotency and the ability to clone in vitro. Applied regeneration protocol for *H. perforatum*, based on ½ MS medium supplemented with 0.45 μM thidiazuron (TDZ), turned out to be an innovative and more effective technique for hyperforin sourcing compared to the harvesting of plants from nature. The ability of TDZ to improve hyperforin production has been reported for *H. hirsutum* [43]. Taking into account anti-cancer activity of some *H. perforatum* secondary metabolites that occurred in different compositions of plants and plant extracts, there is a strong need to study the effect of extracts with different chemical compositions, including those obtained from in vitro cultures, on tumor cells.

### 3.2. Hypericum perforatum Extracts and Hyperforin Salt Have a Different Effect on Cell Viability and Apoptosis in Primary and Metastatic Melanoma

The cytotoxicity of almost all extracts and hyperforin salt was evidenced in normoxia, with the best effect (the lowest IC50) of in vitro cultured St. John’s wort extract (HpEx13), containing the highest content of hyperforin in dry mass in both cell lines tested. Under hypoxic conditions, the strongest cytotoxic action (the lowest IC50) was observed for commercial extract (HpEx2) in both primary and metastatic cell lines, suggesting that despite trace amounts of hyperforin, the anti-proliferative/growth inhibition/cytotoxic effect is visible and can be connected with a high phenolic content in dry mass [44,45,46]. Interestingly, metastatic cells showed an increase in viability in hypoxia after 24 h of incubation with HpEx12 and HpEx13, and this effect was also visible in WM266-4 treated with hyperforin salt, but not in the WM266-4 cells treated with hyperforin-poor HpEx2A. Pure hyperforin salt has a weaker pro-apoptotic effect in metastatic cells, both under hypoxic and normoxic conditions. These observations may suggest the antagonistic, promoting effect of hyperforin (its high amount was noted in HpEx12 and Hpx13) and the inhibitory and pro-apoptotic properties of phenols and other Hp compounds, including hypericin, on cell viability [47,48]. Hypericin has been shown to have light-dependent and light-independent effects on cell viability in several human cancer cell lines [49]. It is also known that hypericin triggers apoptosis, necrosis, or autophagy in melanoma, depending on the presence of a pigmented phenotype in cells [50]. What is more, the pro-apoptotic effect exerted by extracts is greater than the effect of a pure hyperforin salt, due to the activity of flavonoids (a group of polyphenols) inducing intrinsic and extrinsic apoptotic pathways, which can also suppress cell proliferation by inhibiting the epidermal growth factor receptor (EGFR) [51]. Several studies describe the effect of *H. perforatum* on melanoma cells [52,53,54] and suggest the use of hypericin as a cytotoxic agent in photodynamic therapy. The mechanism of hyperforin-dependent apoptosis induction in hepatocellular carcinoma cells involved the enhancement of caspase activity, loss of mitochondrial membrane potential, and decrease in anti-apoptotic proteins [26,29]. Furthermore, combined hyperforin–paclitaxel treatment promoted growth inhibition and apoptosis in human breast cancer cells [31]. At the same time, there are no clear reports showing the effect of hyperforin on melanoma cells. Extremely important is that hypoxia enhances the proapoptotic effect of extracts on primary and metastatic melanoma, but does not enhance the effect of hyperforin salt on any cell line. Hypoxic conditions have important effects on apoptosis, including hypoxia-inducible factor 1 (HIF-1)-mediated up-regulation of pro-apoptotic proteins (such as BNIP3), increased permeability of the inner mitochondrial membrane, and the release of cytochrome c. However, there are antagonistic mechanisms, including the down-regulation of the pro-apoptotic Bax protein by an inhibitor of apoptosis protein 2 (IAP-2). In solid tumors, the phenomenon of hypoxia is frequent, and in aggressive cancers, hypoxia-induced resistance to apoptosis commonly appears [55]. In hypoxic WM266-4 cells, the hyperforin salt-dependent mechanism can act by up-regulating anti-apoptotic pathways.

### 3.3. Hypericum perforatum Extracts and Hyperforin Salt Regulate the Melanoma Oxidative Stress Response Pathway Differently in Hypoxia and Normoxia

As confirmed by the NBT assay, both the extracts and the hyperforin salt, after 24 h of treatment, slightly decrease ROS production under both hypoxia and normoxia conditions. The significance of this effect is evident in cells treated with the highest doses of extracts. The particularly strong antioxidative effect of HpEx2 (the most polyphenol-rich and with the highest DPPH-scavenging activity) may be associated with the phenolic antioxidant activity, based on free radical scavenging. Reports suggest that hyperforin has a weak effect on DPPH inhibition, but some phenolic compounds, such as *p*-cumaroylquinic acids, quercetin, rutin, adhyperforin, and some procyanidin derivatives, are probably responsible for the majority of DPPH scavenging, like in other *Hypericum* species [36,56]. In the studied HpExs, DPPH scavenging activity correlates with total phenolic compounds, similar to the results obtained by Marreli et al. [45]. Phenolic compounds, despite their hydrophilic properties, can be taken up by the cell [57], therefore, they can scavenge cellular free radicals. In contrast, reduction of the signal in the NBT assay may be the result of reduced viability of cells treated with the highest concentrations of extracts. Taking into account both mechanisms, we speculate that there is a synergistic (cytotoxic and intracellular ROS-reduction) mechanism of action of HpEx2. Interestingly, a similar trend of decreasing ROS is revealed in the case of hyperforin salt, but it can act in different ways, as described by Menegazzi et al. [58]. Hyperforin, with hydrophobic and weak cationic properties, enters the cell, locates the mitochondrial inner membrane, and acts as a protonophore, protecting the cell against endogenous ROS production, which may additionally inhibit cell proliferation. In addition, isolated hyperforin is less stable than hyperforin in extracts, where it is protected by other natural antioxidant components. Therefore, it seems that the better solution for research aimed at drug selection is the use of more stable hyperforin derivatives (such as hyperforin salts). In this study, hyperforin salt led to a decrease in intracellular ROS (NBT assay) after 24 h of treatment, but it did not have a free radical scavenging effect (DPPH assay, data not shown), while having a promoting effect on the growth of WM266-4 cells in hypoxia at the same time, which can be seen as no reduction in viability.

Dose-dependent increases in NRF2 and HO-1 levels after 24 h incubation was observed for both HpEx2 and HpEx12. NRF2, a critical regulator of oxidative stress, induced by high cellular ROS [10], can also be activated by various chemical factors through the p53-p21 signaling pathway, leading to DNA damage repair and cell protection [59], which theoretically suppresses cell mutation and tumor evolution. On the other hand, the high level of this transcription factor and its target genes encoding cytoprotective proteins (such as *HMOX1* encoding HO-1) can protect tumor cells against chemotherapy. Interestingly, many cancer cell lines overexpress NRF2 [60,61,62]. The NFR2–HO-1 axis may affect other cellular signaling pathways, e.g., overexpression of NRF2 can be connected to EGFR-dependent proliferation [12]. It can induce the antiapoptotic Bcl-2 protein, resulting in a decrease in the level of proapoptotic Bax and caspase 3/7, and, finally, promote cell defense against apoptosis [63]. Additionally, HO-1 may promote tumor metastasis and malignancy [14,64]. The dualistic role of NRF2 and HO-1 depends on the cell line, the stage of progression, and oxygen level conditions [62]. These complicated, multidimensional, and pleiotropic effects, may explain the different regulation of *NFE2L2* and NRF2 expression by hyperforin salt. Our study reveals that the level of HO-1 decreases depending on the dose of hyperforin salt. This protein encoded by *HMOX1* confers cytoprotection against oxidative stress [64]. In connection with the induction of apoptosis, this single metabolite can provide hope for the treatment of primary melanoma, however, resistance of the metastatic line, no induction of apoptosis, and no decrease in cell viability make the use of hyperforin and its derivatives ineffective in potential metastatic chemotherapy. Overall, at the gene expression level we observed the difference depending on the dose and cell line in normoxic and hypoxic conditions. The obtained results show the enormous complexity of the mechanisms of action of the extracts used, depending on their composition, and also prove that hyperforin and its derivatives as single molecules can act differently, activating various signaling pathways in the cell.

## 4. Materials and Methods

### 4.1. Plant Material and Ethanolic Extraction of Hypericum perforatum

For this study, after preliminary experiments, we selected three *H. perforatum* extracts: HpEx2, HpEx12 (self-made), and HpEx13 (commercially available). The plants for extraction were collected from two wild populations growing on meadows located in Krakow, with exact coordinates: 50° 01′37″N 19° 52′59″E, 208 m a.s.l. and 50° 01′37″N 19^°^ 51′52″E, 205 m a.s.l. The plants were kindly identified by a systematic botanist from Jagiellonian University, Agnieszka Nobis PhD, DSc. The aerial parts of mature *H. perforatum* plants for HpEx12 were harvested in July 2021. To receive HpEx13, we used in vitro cultured regenerants obtained by indirect organogenesis from callus tissue derived from leaves and internodes explants. The plant material for this extract was collected from the same population in September 2020. Regeneration was performed under sterile conditions, using standard ½ Murashige&Skooge (MS) medium [65] supplemented with 0.45 μM thidiazuron (TDZ) (Merck), a synthetic cytokinin-like plant growth regulator, and afterwards 1–2 cm high regenerants were cut off callus and rooted in ½ MS medium. Cultures were stored at 23 ± 2 °C in photoperiod conditions with 16-h irradiation and 8-h darkness, and a red-blue light intensity of 39 µmol/m^2^⋅s. Individuals above 20 cm height from wild populations and in vitro regenerants were collected, lyophilized, and extracted with 99% ethanol in the Soxhlet extractor, and then concentrated approximately twice by evaporation of the solvent (Supplementary Materials Table S2). All extracts were filtered through a 0.22 μm membrane filter (99722, TPP, Switzerland, Trasadingen), partitioned into Eppendorf tubes, and stored in the dark at −20 °C until cell stimulation.

### 4.2. Evaluation of the Ethanol-Solved Compounds Concentration in Extracts

To determine the concentration of ethanol-solved compounds, the solvent was evaporated under vacuum conditions (CentriVap Concentrator, Labconco) from a known volume of the extracts and the concentration (*w*/*v*) was calculated by weighting the mass of solid ethanol-solved compounds on an analytical balance (PRL TA 13, MERA-WAG, Gdansk, Poland). The extracts were adjusted to a concentration of 3 mg/mL and used for analytical tests 4.4–4.6.

### 4.3. HPLC-MS/MS Analysis of Hyperforin Content in H. perforatum Extracts

The hyperforin content in HpEx2, HpEx12 and HpEx13 was analyzed using high-performance liquid chromatography coupled with a tandem mass spectrometer (HPLC-MS/MS). An Agilent Infinity 1260 (Agilent Technologies, Waldbronn, Germany) chromatograph hyphened with an Agilent 6410 (Agilent Technologies, Santa Clara, CA, USA) was used. Chromatographic separation was done on Poroshell 120 SB-C18 2.1 × 30 mm, 2.7 um (Agilent Technologies, Santa Clara, CA, USA) in the gradient mode of A) H_2_O and B) ACN, with 0.1% formic acid. The gradient started at 65% B and ended at 100% B in 5 min, with a flow rate of 0.5 mL min, at 40 °C. Two pairs of precursor and daughter ions were monitored for each compound (hyperforin 537.4 → 277.2 and 537.4 → 203.1) in multiple reaction-monitoring modes (MRM) at positive electrospray ionization (ESI+). For quantification, external calibration was applied using a pure hyperforin dicyclohexylammonium salt standard (H1792, Sigma-Aldrich, St. Louis, MO, USA).

### 4.4. Total Phenolic Content (TPC)

For the assessment of the polyphenol content in extracts, a TPC colorimetric assay based on Saddiqe et al. [66] procedure (modified) was conducted for compounds with reducing properties. The concentrations of extracts tested in TPC were: 3, 1.5, 0.75 and 0.375 mg/mL. As a standard for the determination of the calibration curve, a gallic acid (GA, 398225, Sigma-Aldrich) water solution in concentrations: 0, 0.0275, 0.055, 0.0825, 0.11, 0.165, 0.22, 0.275, 0.33 mg/mL GA/30 mL was used as a standard. The test has been performed using a 96-well plate, according to the following procedure: 100 μL of analytically pure deionized water was added to each well. In the next step, 30 μL of extract/GA solution per well was added. Subsequently 60 μL of Na_2_CO_3_ solution saturated in 4 °C, brought to room temperature, was added to each well and next 50 μL of Folin–Ciocâlteu reagent diluted 1:5 with water was also added per well. The plate was incubated in darkness for 15 min at room temperature, then shaken for 30 s, and the absorbance was measured at 725 nm in 25 °C. Three chemical repetitions were performed for each extract concentration. The total content of the phenolic compounds was expressed as an equivalent of GA (GAE/dry extract mass).

### 4.5. DPPH Radical Scavenging Assay

The antioxidant activity of the extracts was measured by colorimetric DPPH (2,2-diphenyl-1-picryl hydrazyl) (D9132, Sigma-Aldrich) assay according to Saddiqe et al. [66]. The concentrations of extract used for the analysis were: 0.375, 0.75, 1.5 and 3 mg/mL. The hyperforin salt was also used, in the same amount as hyperforin occurring in HpEx12, to determine its radical scavenging activity. A calibration curve was determined using GA (398225, Sigma-Aldrich-) 0, 27.5, 55 and 82.5 µg/mL water solutions. The DPPH test was performed on a 96-well plate. To 95 μL, 300 μM DPPH ethanolic and 5 μL of extract/GA solutions were added. The plate was covered with parafilm and incubated overnight at room temperature in the darkness. The absorbance was measured at 515 nm. The effect was visible as decolorization (from purple to yellow). The radical scavenging activity was shown as the IC50 of DPPH.

### 4.6. In Vitro Culture and Treatment of Melanoma Cells

Two human melanoma cell lines, established from a 55-year-old female at different tumour stages, were supplied by the ESTDAB Melanoma Cell Bank (Tübingen; Germany). WM115 primary melanoma cells represent the vertical growth phase (VGP) and WM266-4 line was derived from lymph node metastasis. Hs27 human skin fibroblast line was purchased from the European Collection of Authenticated Cell Cultures (ECACC, UK) and the line was kindly given by the Chair of Medical Biochemistry, Faculty of Medicine, Jagiellonian University Medical College in Krakow (Poland).

Cells were maintained in RPMI 1640 medium with GlutaMAX-I (72400-021, Gibco, Paisley, UK) supplemented with 10% FBS (10270-106, Gibco) and antibiotics (100 U/mL penicillin and 100 µg/mL streptomycin; 15140-122, Gibco). Cells were grown in monolayers in an atmosphere of 5% CO_2_ in 21% O_2_ (normoxia) at 37 °C, in a humidified incubator Forma Steri-Cycle i160 (Thermo Fisher Scientific, Rockford, IL, USA). Cells were passaged after reaching approximately 80% confluence. Cells were *Mycoplasma*-free, as determined regularly using the MycoAlertTM Mycoplasma Detection Kit (LT07-418, Lonza, Basel, Switzerland).

Melanoma cells (2–10 passages after unbanking) were disseminated into 96-well plates at a density of 3 × 10^4^ cells/well (MTT, NBT), as well as into 60 mm (flow cytometry, RNA isolation) and 100 mm (protein extraction) cell culture plates at a density of 0.8 × 10^6^ and 1.2 × 10^6^ cells per plate, respectively. The following day, *H. perforatum* extracts (HpExs 12 and 13), prepared as described in Section 4.1, the commercially available ethanolic extract (HpEx2, Herbapol, Poland), and the hyperforin salt (H1792, Sigma-Aldrich) were diluted in the culture medium and added to cells. The doses of extracts were selected according to Roscetti et al. [67]. Cells were also treated with 96% ethanol in the volume corresponding to the highest doses of extracts to determine the effect of the diluent on cell viability, apoptosis, and oxidative stress (control). The plates were kept under normoxia (21% O_2_, Forma Steri-Cycle i160, Thermo Fisher Scientific) and hypoxia (0.5% O_2_, O_2_ Control Glove Box for Tissue Culture, Coy Laboratory Products, Grass Lake, MI, USA) conditions for 24 h.

### 4.7. MTT Cell Viability Assay

After removal of the culture medium, a 0.5% aqueous thiazolyl blue tetrazolium bromide (MTT) solution (M2128, Sigma-Aldrich) diluted 1:9 in PBS (P4417, Sigma-Aldrich) was added to each well and incubated for 2 h at 37 °C. Acidic isopropanol was then added and cells were incubated overnight at room temperature. The absorbance was measured at 570 nm against a reagent blank. Results were expressed as a percentage of untreated controls, and IC50 values (the concentration of HpExs or hyperforin salt that caused a 50% reduction in cell viability) for each extract and hyperforin salt were calculated.

### 4.8. Apoptosis Detection by Flow Cytometry

#### 4.8.1. Annexin V Assay

FITC Annexin V Apoptosis Detection Kit I (556547, BD Biosciences, San Diego, CA, USA) was used to label human melanoma cells with annexin V-FITC. Cells collected by trypsinization were counted (TC-10, Bio-Rad, Hercules, CA, USA) and washed twice with cold PBS (1100 rpm, 5 min, 4 °C) and resuspended in 100 μL of 1× binding buffer. In the next step, 2.5 μL of annexin V was added and incubated for 15 min at room temperature away from a light source. After 15 min, 400 μL of 1× binding buffer was added to the cell suspension and the incubation was carried out on ice in the dark. Fluorescence intensity was detected for 10^4^ cells per sample using a FACSCalibur flow cytometer (BD Biosciences, San Diego, CA, USA). The percentage of early apoptotic cells was analyzed using the FlowJo software (BD Bioscience, San Diego, CA, USA).

#### 4.8.2. Caspase 3/7 Activity

The CellEvent™ Caspase 3/7 Green Flow Cytometry Assay Kit (C10740, Invitrogen, Thermo Fisher Scientific, Waltham, MA, USA) was used to detect an activity of caspases 3 and 7 in WM115 and WM266-4 cells treated with *H. perforatum* or hyperforin salt. Cells collected by trypsinization were centrifuged, resuspended in 0.5 mL of PBS, and counted (TC-10, Bio-Rad). Then 0.5 μL of CellEvent® Caspase-3/7 Green Detection Reagent was added to 10^4^ cells in each sample and incubated for 30 min at 37 °C in the dark. Fluorescence intensity was measured for 10^4^ cells per sample in triplicate in the FACSCalibur flow cytometer. The percentage of cells with active caspase 3/7 was determined using the FlowJo software (BD Bioscience, San Diego, CA, USA).

### 4.9. NBT Test

ROS level was determined by an NBT test according to Mazur-Biały [68]. Briefly, after harvesting the culture medium, a 4-nitro blue tetrazolium chloride (NBT) solution (10 mg/mL; N6876, Sigma-Aldrich) was added to each well, cells were incubated for 90 min and then fixed with methanol for 15 min. The plate was thoroughly dried and 70 μL dimethylsulphoxide (DMSO; A3672-0050, PanReac AppliChem) and 60 μL 2 M potassium hydroxide (746800113, POCH, Gliwice, Poland) were added to extract the dye. The absorbance was recorded at 620 nm against a reagent blank. The results were standardised against untreated cells.

### 4.10. Gene Expression Analysis

#### 4.10.1. RNA Isolation

Total RNA was extracted from melanoma cells using a modified Chomczynski method [69]. Cells were lysed with 400 μL TRI Reagent^®^ (T9424, Sigma-Aldrich) and then extracted with 100 μL chloroform (234431116, POCH, Gliwice, Poland). The samples were thoroughly vortexed, incubated for 20 min on ice and centrifuged (10,000 rpm, 20 min, 4 °C). In the next step, the aqueous phase was collected (400–500 μL) and an equivalent amount of isopropanol (603-117-00-0, Stanlab, Lublin, Poland) was added to the precipitate RNA. After 2 h 30 min of incubation, the samples were centrifuged at 10,000 rpm for 30 min at 4 °C. The supernatant was then extracted from above the RNA pellet, which was washed twice with 70% ethanol (603-002-00-5, Stanlab, Lublin, Poland) and centrifuged (10,000 rpm, 10 min, 4 °C). After ethanol removal, the pellets were allowed to dry. RNA was resuspended in 10–15 μL RNase free water and incubated at 65 °C for 8 min. RNA quality and concentration were determined spectrophotometrically (NanoDrop 2000, Thermo Fisher Scientific) at 260 nm and 280 nm against RNase-free water. The isolated RNA was stored at −80 °C.

#### 4.10.2. cDNA Synthesis

The High-Capacity RNA-to-cDNA Reagent Kit (4387406, Applied Biosystems, Foster City, CA, USA) was used to transcribe mRNA into complementary DNA by reverse transcription. A 10:1 mixture of reaction buffer and reverse transcriptase was prepared and 5.5 μL of the mixture was added to each sample containing 0.5 μg of RNA in 4.5 μL RNase-free water. The solution was placed in a thermoblock (Eppendorf), and the reaction was carried out at 37 °C for one hour. The temperature was then increased to 95 °C for 5 min. The contents of the tubes were cooled to 4 °C, then frozen at −20 °C and stored until RT-qPCR analysis.

#### 4.10.3. RT-qPCR

qRT-PCR reactions were performed using Power SYBR Green PCRMaster Mix (4367659, Applied Biosystems) and the specific primers for HMOX1 (F: 5′-AGTGTAAGGACCCATCGGAG-3′ and R: 5′-CATGACACCAAGGACCAGAG-3′) and NFE2L2 (F: 5′-TTGAGCAAGTTTGGGAGGAGCTA-3′ and R: 5′-GGAGAGGATGCTGCTGAAGG-3′) (Genomed, Warsaw, Poland) in QuantStudioTM Design & Analysis Software v1. 5.1 (Thermo Fisher Scientific). Gene encoded hypoxanthine phosphoribosyltransferase 1, HPRT (F: 5′-CATTATGCTGAGGATTTGGAAAGG-3′ and R: 5′-CTTGAGCACACAGAGGGCTACA-3′) (Genomed, Warsaw, Poland) served as a housekeeping gene. The RT-qPCR data were quantified by the 2^−∆∆Ct^ method.

### 4.11. Western Blotting

Total homogenates from melanoma cell lines were obtained using RIPA buffer (89900, Thermo Fisher Scientific, Waltham, MA, USA) containing a protease inhibitor mixture (P8340, Sigma-Aldrich). Protein concentration was determined using a Total Protein Kit Micro Lowry Peterson’s Modification (TP0300-1KT, Sigma-Aldrich). Protein samples, containing 20 µg of proteins, were separated under reducing conditions in SDS-PAGE electrophoresis. The separated protein was then electrotransferred onto a PVDF membrane (88518, Thermo Fisher Scientific, Waltham, MA, USA). Non-specific binding sites on PVDF membranes were blocked overnight in 1% BSA at 4 °C (β-actin, ACTB) or 5% skim milk in TBST at room temperature (HO-1 and NRF2). In the next step, membranes were incubated with rabbit anti-HO-1 (ADI-SPA 894, Enzo, NY, USA), rabbit anti-NRF2 (16396-1-AP, Proteintech, Rosemont, IL, USA) or mouse anti-β-actin (A5441, Sigma-Aldrich) primary antibody overnight (anti-HO-1 diluted 1:2000 and anti-NRF2, diluted 1:1000) at 4 °C, or 1 h (anti-β-actin, diluted 1: 10 000) at room temperature. After rinsing three times with TBST, the membranes were incubated with horseradish peroxidase-conjugated secondary antibody, goat anti-rabbit IgG (AP307P, Millipore, Burlington, MA, USA) diluted 1:5000 or goat anti-mouse IgG (7076, Cell Signaling Technology, MA, USA), diluted 1:4000, for 1 h at room temperature. Specific protein bands were visualized by chemiluminescence after an addition of Immobilon Western Chemiluminescent HRP Substrate (WBKLS, Millipore, Burlington, MA, USA) in ChemiDoc™ XRS+ Imaging System (Bio-Rad).

### 4.12. Statistical Analysis

All experiments were performed in triplicate or duplicate, and within one experiment, three technical repetitions for each sample were done. The results are represented as means ± SD. Statistical analyses were performed using GraphPad Prism 9. Statistical significance for the data was evaluated by a one- (MTT test for Hs27) and two-way (other experiments) analysis of variance (ANOVA), followed by a Tukey’s test of multiple differences. A *p* value < 0.05 was considered statistically significant.

## 5. Conclusions

Our study showed that oxygen metabolism and the NRF2/HO-1 signaling pathway in human melanoma cells may be regulated by secondary metabolites extracted from *H. perforatum* and hyperforin salt. Hyperforin and its more stable derivatives are interesting chemicals for cancer treatment due to their proapoptotic and antioxidative properties. The response of cutaneous melanoma cells, especially the WM266-4 metastatic cell line, to the active compounds in *H. perforatum* extracts depended on hypoxic and normoxic conditions. The synergistic effects of variable compounds, especially bioactive phenolic compounds and hyperforin, present in the *H. perforatum* extracts, or the effect of pure hyperforin in the salt form are crucial for the viability, apoptosis, and oxidative stress of melanoma cells. The salient finding of our study is that a source for efficient extraction and purification of hyperforin from raw material is important. Our extracts from wild-growing *H. perforatum* and plants regenerated on the basis of the same wild populations differed significantly in the content of hyperforin from the commercially available extract. In vitro *H. perforatum* regenerants were a more valuable source of hyperforin than wild-growing plants, which was the result of culture conditions (thidiazuron application).

To sum up, our study showed that the anticancer activity of *H. perforatum* extracts differs in normoxia and hypoxia, for the first time. Importantly, the composition of extracts of various origins, including in vitro cultured resulting in their unique properties, may be important in the selection of plants for therapeutic application.

## Figures and Tables

**Table 1:** The content of selected secondary metabolites and the radical scavenging activity of the ethanolic extracts of *Hypericum perforatum*. The values obtained for the extracts used in the DPPH assay were adjusted to a concentration of 3 mg/mL.

HpEx	Concentration of Freeze-Dried Raw Material [mg/mL]	Concentration of Ethanol-Solved Compounds [mg/mL]	Hyperforin Content in Extract [%]	Polyphenol Content in Extract [%] TPC Assay	DPPH Radical Scavenging Assay [IC50_DPPH_, μg/mL]
2	n/a	36.0	0.005	9.98 ± 0.75	708.89 ± 29.07
12	271.2 mg/mL	16.4	0.456	8.87 ± 0.52	858.97 ± 5.43
13	285.6 mg/mL	15.0	0.679	8.85 ± 0.47	829.02 ± 22.99

## Data Availability

Not applicable.

## Supplementary Materials

The following supporting information can be downloaded at: https://www.mdpi.com/article/10.3390/molecules28031509/s1, Supplementary Figure S1: Effect of *Hypericum perforatum* extracts HpEx2 (A), HpEx12 (B), and HpEx 13 (C) on the viability of Hs27 human skin fibroblasts cultured under normoxia, determined by MTT assay; Supplementary Table S1: The content of selected secondary metabolites in the volumes of *Hypericum perforatum* ethanolic extracts used to cell assays.
