# Supplementary material for: Cytotoxic and Antioxidant Activity of Hypericum perforatum L. Extracts against Human Melanoma Cells from Different Stages of Cancer Progression, Cultured under Normoxia and Hypoxia
Source: Molecules. 2023 Feb 3;28(3):1509. doi: 10.3390/molecules28031509 (PMC9921514; doi:10.3390/molecules28031509)
Supplement: Supplementary file 1 [file molecules-28-01509-s001.zip › molecules-2168099-supplementary.pdf]

Supplementary Material

Cytotoxic and antioxidant activity of *Hypericum perforatum* L. extracts against human melanoma cells from different stages of cancer progression, cultured in normoxia and hypoxia conditions

Aleksandra Brankiewicz [1,#], Sara Trzos [2,#], Magdalena Mrożek[2], Małgorzata Opydo[3], Elżbieta Szostak[4], Michał Dziurka[5], Monika Tuleja[1], Agnieszka Łoboda[6] and Ewa Pocheć [2,*]

[1] Department of Plant Cytology and Embryology, Institute of Botany, Faculty of Biology, Jagiellonian University, 30-387 Kraków, Poland
[2] Department of Glycoconjugate Biochemistry, Institute of Zoology and Biomedical Research, Faculty of Biology, Jagiellonian University, 30-387 Kraków, Poland
[3] Laboratory of Experimental Hematology, Institute of Zoology and Biomedical Research, Faculty of Biology, Jagiellonian University, 30-387 Krakow, Poland
[4] Faculty of Chemistry, Jagiellonian University, 30-387 Kraków, Poland
[5] The Franciszek Górski Institute of Plant Physiology, Polish Academy of Sciences, 30-239 Kraków, Poland
[6] Department of Medical Biotechnology, Faculty of Biochemistry, Biophysics and Biotechnology, Jagiellonian University, 30-387 Kraków, Poland
[#] These authors contributed equally to this work.
[*] Correspondence: ewa.pochec@uj.edu.pl; Tel.: +48 12 664 64 67

2.2. Hypericum perforatum extracts and hyperforin salt affect melanoma cell viability

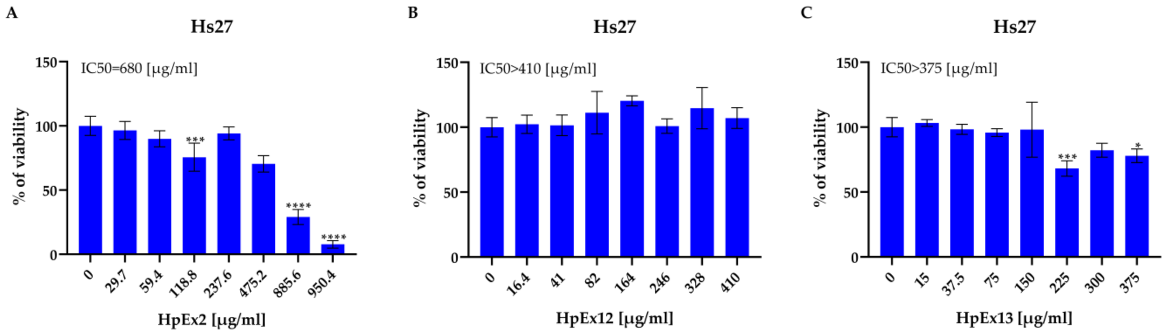

Supplementary Figure S1. Effect of *Hypericum perforatum* extracts HpEx2 (**A**), HpEx12 (**B**), and HpEx 13 (**C**) on the viability of Hs27 human skin fibroblasts cultured under normoxia, determined by MTT assay. Results are expressed as mean values ± SD. IC50 values are shown above the bars. Statistical significance of the data was assessed using one-way ANOVA followed by Tukey's test for honest significant difference in multiple ranges. Significance levels between treated cells relative to untreated cells are indicated with asterisks as follows $*p \leq 0.05$; $**p \leq 0.01$; $***p \leq 0.001$; $****p \leq 0.0001$.

Supplementary Table S1. The content of selected secondary metabolites in the volumes of *Hypericum perforatum* ethanolic extracts used to cell assays. Applied doses marked as C1 – C7.

Extract No.		C1	C2	C3	C5	C6	C7
HpEx2	Volume of extract per well [µl]	0.0825	0.165	0.33	1.32	2.46	-
	Mass of extract per well [µg/0.1 ml]	2.97	5.94	11.88	47.52	88.56	-
	Mass of polyphenols [µg/0.1ml]	0.2964	0.5928	1.1856	4.7425	8.8383	-
	Mass of hyperforin per well [µg/0.1ml]	0.00016	0.00032	0.00064	0.00255	0.00475	-
HpEx12	Volume of extract per well [µl]	0.1	0.25	0.5	1.5	2	2.5
	Mass of extract per well [µg/0.1 ml]	1.64	4.1	8.2	24.6	32.8	41
	Mass of polyphenols [µg/0.1ml]	0.1455	0.3637	0.7273	2.1820	2.9093	3.6367
	Mass of hyperforin per well [µg/0.1ml]	0.00748	0.0187	0.0374	0.11221	0.14962	0.18702
HpEx13	Volume of extract per well [µl]	0.1	0.25	0.5	1.5	2	2.5
	Mass of extract per well [µg/0.1 ml]	1.5	3.75	7.5	22.5	30	37.5
	Mass of polyphenols [µg/0.1ml]	0.1328	0.3319	0.6638	1.9913	2.655	3.3188
	Mass of hyperforin per well [µg/0.1ml]	0.01019	0.02549	0.05096	0.15287	0.20382	0.25478

4.1. Plant material and ethanolic extraction of Hypericum perforatum

Supplementary Table S2. Procedure of *Hypericum perforatum* ethanolic extract preparation.

	Extract No.	**HpEx12**		**HpEx13**	
Step 1	Raw material source	*H. perforatum* wild population 1	*H. perforatum* wild population 2	*H. perforatum* regenerants cultured from population 1	*H. perforatum* regenerants cultured from population 2
	Dry mass of lyophilised raw material	34.7 g	33.1 g	36.4 g	35.0 g
	Volume of ethanol used to extraction in Soxhlet extractor	250 ml	250 ml	250 ml	250 ml
Step 2	Volume of evaporated ethanol	about 125 ml	about 125 ml	about 125 ml	about 125 ml
Step 3	Estimated concentration of finally obtained extracts (used for cell stimulation)	67.8 g / 250 ml = 271.2mg / ml		71.4 g / 250 ml = 285.6 mg / ml	
Step 4	Extraction efficiency assessment	$\frac{16.4\ mg/ml}{271.2\ mg/ml}$= 6,04%		$\frac{15\ mg/ml}{285.6\ mg/ml}$= 5,25%	
